# Evidence-based smoking cessation treatment: a comparison by healthcare system

**DOI:** 10.1186/s12913-020-06016-5

**Published:** 2021-01-07

**Authors:** Jennifer A. Lewis, Nicole Senft, Heidi Chen, Kathryn E. Weaver, Lucy B. Spalluto, Kim L. Sandler, Leora Horn, Pierre P. Massion, Robert S. Dittus, Christianne L. Roumie, Hilary A. Tindle

**Affiliations:** 1 Veterans Health Administration-Tennessee Valley Healthcare System Geriatric Research, Education and Clinical Center (GRECC), Nashville, TN USA; 2 Division of Hematology/Oncology, Department of Medicine, Vanderbilt University Medical Center, 2525 West End Ave., Suite 1200, Nashville, TN 37203 USA; 3 Vanderbilt-Ingram Cancer Center, Nashville, TN USA; 4 Department of Medicine, Vanderbilt University Medical Center, Nashville, TN USA; 5 Department of Biostatistics, Vanderbilt University Medical Center, Nashville, TN USA; 6 Departments of Social Sciences and Health Policy and Implementation Science, Wake Forest School of Medicine, Winston-Salem, NC USA; 7 Department of Radiology, Vanderbilt University Medical Center, Nashville, TN USA; 8 Division of Allergy, Pulmonary, and Critical Care Medicine, Vanderbilt University Medical Center, Nashville, TN USA; 9 Medicine Service, Veterans Health Administration-Tennessee Valley Healthcare System, Nashville, TN USA; 10 Division of General Internal Medicine and Public Health, Department of Medicine, Vanderbilt University Medical Center, Nashville, USA

## Abstract

**Background:**

A systems-level approach to smoking cessation treatment may optimize healthcare provider adherence to guidelines. Institutions such as the Veterans Health Administration (VHA) are unique in their systematic approach, but comparisons of provider behavior in different healthcare systems are limited.

**Methods:**

We surveyed general medicine providers and specialists in a large academic health center (AHC) and its affiliated VHA in the Mid-South in 2017 to determine the cross-sectional association of healthcare system in which the provider practiced (exposure: AHC versus VHA) with self-reported provision of evidence-based smoking cessation treatment (delivery of counseling plus smoking cessation medication or referral) at least once in the past 12 months (composite outcome). Multivariable logistic regression with adjustment for specialty was performed in 2017–2019.

**Results:**

Of 625 healthcare providers surveyed, 407 (65%) responded, and 366 (59%) were analyzed. Most respondents practiced at the AHC (273[75%] vs VHA 93[25%]) and were general internists (215[59%]); pulmonologists (39[11%]); hematologists/oncologists (69[19%]); and gynecologists (43[12%]). Most respondents (328[90%]) reported the primary outcome. The adjusted odds of evidence-based smoking cessation treatment were higher among VHA vs. AHC healthcare providers (aOR = 4.3; 95% CI 1.3–14.4; *p* = .02). Health systems differed by provision of individual treatment components, including smoking cessation medication use (98% VHA vs. 90% AHC, *p* = 0.02) and referral to smoking cessation services (91% VHA vs. 65% AHC *p* = 0.001).

**Conclusions:**

VHA healthcare providers were significantly more likely to provide evidence-based smoking cessation treatment compared to AHC healthcare providers. Healthcare systems’ prioritization of and investment in smoking cessation treatment is critical to improving providers’ adherence to guidelines.

**Supplementary Information:**

The online version contains supplementary material available at 10.1186/s12913-020-06016-5.

## Background

Tobacco use remains the leading cause of preventable death in the United States (U.S.) and illnesses associated with cigarette smoking are responsible for over 480,000 deaths annually [1]. “Tobacco Nation” is an area comprising 13 states in the U.S. Midwest and South where the death rates of tobacco-related illnesses, such as stroke and cancer, are especially high [2–4]. In efforts to improve smoking cessation rates at the population level, the U.S. Public Health Service’s Clinical Guideline on Tobacco Use and Dependence recommends a brief intervention using the 5 A’s (Ask, Advise, Assess, Assist, Arrange) for every current smoker at each clinical encounter [5]. This strategy requires all healthcare providers to “Ask” patients their smoking status; “Advise” current smokers to quit; “Assess” smokers’ willingness to quit; “Assist” smokers with cessation; and “Arrange” follow up to re-address smoking cessation efforts [5]. Unfortunately, healthcare provider adherence to smoking cessation treatment guidelines is sub-optimal [6–9]. Clinicians report routinely performing “Ask” (87–100%) and “Advise” (66–95%), but far fewer perform the “Assess” (39–85%), “Assist” (16–64%), and “Arrange” (1–23%) steps [7].

In the “Assist” of the 5 A’s, the most effective evidence-based strategy is to combine counseling with Food and Drug Administration (FDA)-approved smoking cessation medications (smoking cessation medications), including nicotine replacement therapy (gum, lozenges, patch, nasal spray, oral inhaler), varenicline, and bupropion [5]. Clinicians provide smoking cessation counseling as part of the “Assist” in approximately 20% of visits with smokers and provide smoking cessation medications even less often (< 2%) [6]. The reasons for non-adherence may reflect healthcare systems’ local resources [10] and norms, workflows, priorities, patient factors, or healthcare providers’ attitudes [11]. For example, the Veterans Health Administration (VHA), which is the U.S.’s largest, integrated healthcare system that serves 9 million U.S. Veterans [12], has longstanding smoking cessation programs that combine counseling with FDA-approved smoking cessation medications, including clinical reminders as part of workflow and smoking cessation performance measures that are routinely monitored [13–16]. By contrast, academic health centers (AHCs) are affiliated with individual academic institutions and tend to be more varied in the norms, workflow processes, and resources related to smoking cessation treatment. Although a systems-level approach has been widely advocated [17–20], a direct comparison of healthcare providers’ smoking cessation treatment practices between healthcare systems is understudied. Within the context of the VHA’s established systems-level approach to tobacco control, healthcare providers may be more likely to adhere to smoking cessation treatment guidelines. In this context, we tested the hypothesis that rates of evidence-based smoking cessation treatment would be higher among healthcare providers at a single VHA facility compared to its affiliated AHC.

## Methods

### Study design, setting and participants

We conducted a cross-sectional evaluation of self-reported evidenced-based smoking cessation treatment at an AHC and its affiliated VHA. At the time of the study, the AHC had an inpatient smoking cessation consult service and provided smoking cessation treatment within an American College of Radiology-certified lung cancer screening program. The AHC also had several resources in its electronic health record (EHR), such as a smoking cessation medication order set with accompanying reference text, an ambulatory smart set for smoking cessation treatment, and orders for bi-directional eReferral to state quitlines (i.e., a secure electronic closed loop with initial referral from the EHR to the quitline and feedback report from the quitline back to the EHR) [21]. VHA had an outpatient smoking cessation treatment program/clinic, free group smoking cessation classes, free or low-copay smoking cessation medications available to Veterans and access to a national telephone quitline (1–855-QUIT-VET). There was also an annual smoking cessation treatment clinical reminder for VHA primary care providers, and completion of this clinical reminder was tracked as a quality metric.

Details of the questionnaire have been published elsewhere and are summarized here [22]. Between February and May 2017, we surveyed 625 healthcare providers across four specialties (General Internal Medicine, Pulmonology, Hematology/Oncology, and Gynecology) that are primarily responsible for preventive services (General Internal Medicine, Gynecology), care for patients with smoking-related lung disease (Pulmonology) or patients with a history of cancer (Hematology/Oncology) at two institutions (AHC and VHA) and included hospital-based and community-based practices.

Eligible healthcare providers included attending physicians, physicians-in-training, physician assistants, and nurse practitioners who reported providing healthcare services to patients over the age of 50 in the year prior to the study. Healthcare providers were identified using departmental websites, administrative staff and departmental leaders. Questionnaire responses were confidential and unable to be linked to individual respondents. The Institutional Review Boards at the AHC and the VHA approved the study. A waiver of documentation of written consent was granted for the study; consent was implied upon clicking the questionnaire link after reviewing key study information. All respondents were offered the chance to win a $50 gift card upon questionnaire completion.

### Study procedures

The questionnaire was pilot tested by five healthcare providers to evaluate the length of time to completion, survey fatigue, appropriateness of terminology, comprehension, and reaction to questionnaire items. Minor revisions were made to use terminology specific to the VHA.

Healthcare providers were emailed with an invitation to participate in a research study on lung cancer screening and tobacco cessation attitudes and practices via a web-based questionnaire in AHC Research Electronic Data Capture (REDCap) or VHA REDCap [23]. Weekly email reminders were sent to non-responders by the study team (and by departmental leaders in the final weeks), and daily, personalized reminders were sent the final 3 days of the 12-week study period.

### Questionnaire content

#### Primary independent variable: healthcare system

The primary predictor of interest was the healthcare system in which the healthcare provider delivered most of his/her patient care (AHC or VHA).

#### Dependent variable: provision of evidence-based smoking cessation treatment

Healthcare providers reported whether, during the past 12 months, they had provided smoking cessation treatment consisting of: (1) counseling using the 5 A’s (Ask, Advise, Assess, Assist, Arrange), (2) FDA-approved smoking cessation medications (nicotine replacement therapy, varenicline, bupropion), and (3) referral to smoking cessation services. “Counseling” referred to brief, point-of-care interventions that typically take place during clinic visits but could have included more intensive therapy. Healthcare providers were considered to have delivered evidence-based smoking cessation treatment [5, 24] if they responded “yes” to (1) providing smoking cessation counseling *and* FDA-approved smoking cessation medication *or* (2) referring smokers to smoking cessation services that provide smoking cessation medications and counseling (onsite programs or quitline using standard clinical referral models [e.g., Ask, Advise, Connect and Ask, Advise, Refer]) (Supplemental Table 1) [25]. We used this broad primary outcome definition to account for smoking cessation treatment services available within and outside of both healthcare systems and to be inclusive of the variety of specialties represented in the study.

#### Healthcare provider characteristics

Healthcare providers self-reported their gender, race/ethnicity, and several professional characteristics: medical position, years in training/practice, medical specialty, percentage of time in direct patient care, practice setting (hospital-based vs community-based), and percentage of smokers and non-smokers in their practice (Supplemental Table 1). We calculated “years since terminal degree” for each healthcare provider by accounting for medical position and years in training for attending physicians. This was calculated separately for each individual specialty and categorized as < 1 year, 1–5 years, 6–10 years, 11–15 years, 16–25 years, and > 25 years.

Healthcare providers reported how effective they believed smoking cessation treatment is at reducing cancer mortality using a four-point Likert-like scale (1 = Very effective, 4 = Not effective). Attitudes towards smoking cessation treatment were dichotomized as “very effective” versus less than “very effective” (moderately effective, minimally effective, not effective, don’t know).

### Statistical analysis

The analytic sample included all healthcare providers who responded to questionnaire outcome items on provision of evidence-based smoking cessation treatment (counseling plus smoking cessation medication or referral). We first described healthcare provider characteristics by healthcare system and tested whether VHA and AHC healthcare providers differed across baseline characteristics using Pearson Chi-Square tests for categorical and ordinal variables and Wilcoxon Rank Sum test for continuous variables. Unadjusted logistic regressions tested the association between healthcare system (VHA, AHC) and provision of evidence-based smoking cessation treatment. A multivariable logistic regression assessed the association between healthcare system and provision of evidence-based smoking cessation treatment, adjusting for medical specialty given the varying healthcare provider types recruited; the study was under-powered to include additional covariates as potential confounding variables. A secondary analysis, adjusted by healthcare system, used logistic regression to explore the associations between healthcare provider characteristics and provision of evidence-based smoking cessation treatment (outcome); each provider variable was entered into the main multivariable model one at a time. All analyses were conducted using R software version 3.6.1from 2017 to 2020.

## Results

**Fig. 1:**
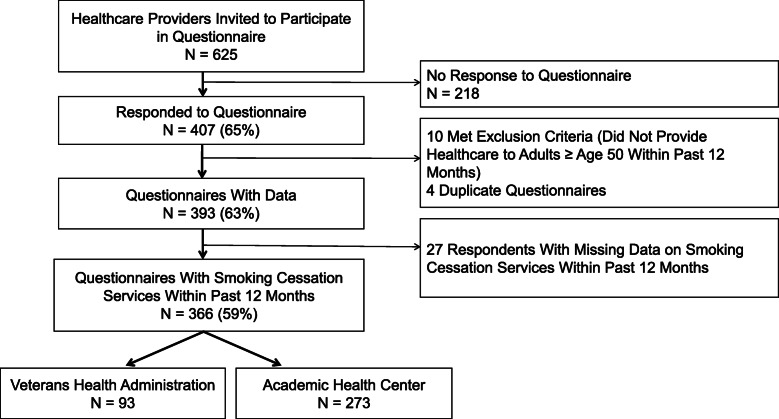
Flow Chart of Questionnaire Participants. legend: *Healthcare Providers included: General Internal Medicine, Pulmonology, Hematology/Oncology, and Gynecology clinicians who practiced in hospital-based and community-based settings

We invited 625 healthcare providers (449 AHC, 176 VHA) to complete the questionnaire and 407 (65%) responded (309 AHC [68.9%], 98 VHA [55.7%]). Questionnaire non-responders, compared to responders, were more likely to be attending physicians (61.0% vs 46.7%) in pulmonology (18.3% vs 10.7%) and to work at VHA (35.8% vs 25.4%) (Supplemental Table 2). Of those who responded, we excluded 10 participants who reported they did not provide healthcare services to adult patients in the year prior to the study. Four respondents with dual AHC and VHA appointments completed the questionnaire twice. All four provided healthcare predominantly at VHA, so their responses in VHA REDCap were included and responses in AHC REDCap were removed. We excluded 27 additional questionnaire responses because those healthcare providers were missing data on the primary outcome, thus the final analytic sample included 366 questionnaires (Fig. 1).

### Characteristics of respondents

Of the respondents in the analytic sample, 273 healthcare providers worked at the AHC (75.6%) and 93 (25.4%) healthcare providers worked at the VHA. Compared to VHA healthcare providers, AHC healthcare providers were more likely to be physicians-in-training (46.9% vs 33.3%), had fewer years in practice (19.4% vs 37.6% with ≥16 years since terminal degree), and were less likely to practice in general internal medicine (54.9% vs 69.6%) or in community-based settings (25.3% vs 37.6% %) (Table 1).

**Table 1:** Characteristics of respondents by healthcare system

Characteristic	Total Sample ***N*** = 366 (%)	VHA^a^ ***N*** = 93 (%)	AHC^b^ ***N*** = 273 (%)
**Sociodemographic Characteristics**			
Gender, n (%)			
Female	206 (56.3)	51 (54.8)	155 (56.8)
Ethnicity/Race, n (%)^c^			
Hispanic	11 (3.0)	2 (2.2)	9 (3.3)
White	297 (81.1)	71 (76.3)	226 (82.8)
Black	22 (6.0)	7 (7.5)	15 (5.5)
Asian	45 (12.3)	15 (16.1)	30 (11.0)
Other	15 (4.1)	4 (4.3)	11 (4.0)
**Professional Characteristics**			
Medical Specialty, n (%)			
General Internal Medicine	215 (58.7)	65 (69.9)	150 (54.9)
Pulmonology	39 (10.7)	10 (10.8)	29 (10.6)
Hematology/Oncology	69 (18.9)	18 (19.4)	51 (18.7)
Gynecology	43 (11.7)	0	43 (15.8)
Medical Position, n (%)			
Attending	171 (46.7)	45 (48.4)	126 (46.2)
Physician-In-Training	159 (43.4)	31 (33.3)	128 (46.9)
Nurse Practitioner	33 (9.0)	17 (18.3)	16 (5.9)
Physician Assistant	3 (0.8)	0	3 (1.1)
Years Since Terminal Degree, n (%)			
< 1	38 (10.4)	5 (5.4)	33 (12.1)
1–5	137 (37.4)	29 (31.2)	108 (39.6)
6–10	54 (14.8)	13 (14.0)	41 (15.0)
11–15	47 (12.8)	11 (11.8)	36 (13.2)
16–25	57 (15.6)	25 (26.9)	32 (11.7)
> 25	31 (8.5)	10 (10.8)	21 (7.7)
Missing	2 (0.5)	0	2 (0.7)
Practice Location, n (%)			
Hospital-based clinic	262 (71.6)	58 (62.4)	204 (74.7)
Community-based clinic	104 (28.4)	35 (37.6)	69 (25.3)
< 50% of Time Providing Direct Patient Care, n (%)	87 (23.8)	26 (28.0)	61 (22.3)
Perceived Percentage of Current Smokers in Healthcare Provider’s Practices, median (IQR)	25 (15, 40)	40 (30, 50)	20 (15, 30)
Perceived Percentage of Former Smokers in Healthcare Provider’s Practices, median (IQR)	30 (20, 50)	40 (25, 70)	30 (20, 40)
Perceived Effectiveness of Smoking Cessation at Decreasing Cancer Mortality, n (%)			
Very Effective	260 (71.0%)	69 (74.2%)	191 (70.0%)
< Very Effective	104 (28.4%)	23 (24.7%)	81 (29.7%)
Missing	2 (0.5%)	1 (1.1%)	1 (0.4%)

^a^VHA refers to Veterans Health Administration, ^b^AHC refers to academic health center, ^c^Healthcare providers were allowed to select all race/ethnicity options that applied

### Primary analysis: evidence-based smoking cessation treatment and healthcare system

In the analytic cohort, 90% of healthcare providers self-reported provision of evidence-based smoking cessation treatment at least once within the past 12 months. Counseling (provided by 74%) and smoking cessation medication (provided by 92%) were common; however only 66% of providers reported providing both counseling and FDA-approved smoking cessation medication, which is the gold-standard recommended by guidelines [5, 26]. Referral to smoking cessation services was reported by 72% of providers. VHA healthcare providers had 4.4 times higher unadjusted odds of providing evidence-based smoking cessation treatment (95% CI 1.3–14.7; *p* = 0.016) compared to AHC healthcare providers (97% VHA vs. 87% AHC). These results were robust to adjustment by specialty (adjusted odds ratio [aOR] 4.3; 95% CI [confidence interval], 1.3–14.4; *p* = 0.020).

We further evaluated differences in individual components of the composite outcome, evidence-based smoking cessation treatment, across healthcare systems. The proportion of healthcare providers who self-reported provision of counseling was similar between healthcare systems (76% VHA vs. 73% AHC). There was a small, statistically significant difference in the self-reported provision of smoking cessation medications between healthcare systems (98% VHA vs. 90% AHC; *p* = 0.020). Healthcare systems differed substantially in their rates of referral to smoking cessation services (91% VHA vs. 65% AHC *p* = < 0.001).

Secondary Analysis: Evidence-based Smoking Cessation Treatment by Baseline Characteristics of Healthcare Providers.

#### Attitudes towards smoking cessation effectiveness

A total of 260 (71%) rated smoking cessation treatment as “very effective” at reducing cancer-related mortality. Attitudes towards smoking cessation treatment were unrelated to provision of evidence-based smoking cessation treatment (Table 2).

**Table 2:** Provision of evidence-based smoking cessation treatment by healthcare system and provider characteristics

Exposure	Provided Evidence-Based Smoking Cessation Treatment ***N*** = 328 (%)^**a**^	Did not Provide Evidence-Based Smoking Cessation Treatment ***N*** = 38 (%)^b^	Adjusted OR (95% CI) of Providing Evidence-Based Smoking Cessation Treatment ^c^
**Primary Analysis: Healthcare System**			
Veterans Health Administration	90 (27.4%)	3 (7.9%)	4.3 (1.3–14.4)
Academic Health Center	238 (72.6%)	35 (92.1%)	Ref^d^
**Secondary Analysis: Healthcare Provider Baseline Characteristics**			
Sociodemographic Characteristics			
Gender, n (%)			
Female	182 (55.5%)	24 (63.2%)	0.7 (0.4–1.5)
Ethnicity/Race, n (%)^c^			
White	261 (79.6%)	36 (94.7%)	0.2 (0.1–1.0)
Non-White	67 (20.4%)	2 (5.3%)	Ref
Professional Characteristics			
Medical Specialty, n (%)			
General Internal Medicine	199 (60.7%)	16 (42.1%)	1.4 (0.4–4.4)
Hematology/Oncology	57 (17.4%)	12 (31.6%)	0.5 (0.2–1.8)
Pulmonology	35 (10.7%)	4 (10.5%)	Ref
Gynecology	37 (11.3%)	6 (15.8%)	0.9 (0.2–3.5)
Medical Position, n (%)			
Attending	156 (47.6%)	15 (39.5%)	Ref
Non-Attending	172 (52.4%)	23 (60.5%)	0.7 (0.4–1.5)
Years Since Completion of Terminal Degree, n (%)			
< 1–5	155 (47.3%)	20 (52.6%)	Ref
6–15	91 (27.7%)	10 (26.3%)	1.1 (0.5–2.5)
> 16	80 (24.4%)	8 (21.1%)	1.1 (0.4–2.6)
Missing	2 (0.6%)	0	
Practice Location, n (%)			
Hospital-based clinic	240 (73.2%)	22 (57.9%)	2.3 (1.1–4.7)
Community-based clinic	88 (26.8%)	16 (42.1%)	Ref
< 50% of Time Providing Direct Patient Care, n (%)	79 (24.1%)	8 (21.1%)	1.1 (0.5–2.6)
Perceived Percentage of Current Smokers in Healthcare Provider’s Practice, median (IQR)	25 (15, 40)	20 (10, 25)	1.3 (1.0–2.7)
Perceived Percentage of Former Smokers in Healthcare Provider’s Practice, median (IQR)	30 (20, 50)	35 (20, 50)	0.9 (0.8–2.7)
Perceived Effectiveness of Smoking Cessation at Decreasing Cancer Mortality, n (%)			
Very Effective	234 (71.3%)	26 (68.4%)	1.1 (0.5–2.3)
Not Very Effective	92 (28.0%)	12 (31.6%)	Ref
Missing	2 (0.6%)	0	

^a^Evidence-based smoking cessation treatment defined as providing counseling and FDA-approved smoking cessation medications or placing a referral for smoking cessation services within the past 12 months. ^b^Non-evidence-based smoking cessation treatment defined as not providing counseling and FDA-approved smoking cessation medications or placing a referral for smoking cessation services within the past 12 months. ^c^For the primary analysis, logistic regression (adjusted by medical specialty) was used to assess the association of healthcare system with the odds of providing evidence-based smoking cessation treatment; for the secondary analysis logistic regression (adjusted for healthcare system) was used to assess the association of healthcare provider characteristics with the odds of providing evidence-based smoking cessation treatment; Missing values were not included in the models. ^d^Ref refers to reference

#### Healthcare provider characteristics

After adjustment by healthcare system, provision of evidence-based smoking cessation treatment was lower among White versus non-White providers (e.g. Hispanic, Black, Asian, Other) (aOR 0.2 [95% CI 0.1–1.0; *p* = 0.048]), in hospital-based clinics than in community-based clinics (aOR 2.3 [95% CI 1.1–4.7; *p* = 0.020]), and among healthcare providers who estimated higher percentages of current smokers in their clinical practice (aOR 1.3 per 10% increase in current smoker estimation [95% CI 1.0–2.7; *p* = 0.038]) (Table 2).

## Discussion

This is the first study to our knowledge that compares healthcare providers’ smoking cessation treatment practices across U.S. healthcare systems. We found that VHA healthcare providers had 4 times the odds of self-reported evidence-based smoking cessation treatment compared to healthcare providers at the AHC. Nearly all (98%) of the healthcare providers at the VHA reported prescribing FDA-approved smoking cessation medications at least once during the past year, with approximately 75% reporting that they provided smoking cessation counseling, and 64% reporting that they provided both counseling and smoking cessation medication (gold standard). These findings mirror national trends in lower-than-ideal rates of provision of both smoking cessation medication and counseling [27]. Rates were slightly lower, though similar, among AHC healthcare providers (90% provided FDA-approved smoking cessation medications, 73% counseling, and 64% both). The difference in overall rate of providing evidence-based smoking cessation treatment was driven by large differences in rates of referrals to smoking cessation services, reported by over 90% of VHA healthcare providers but less than two-thirds of AHC healthcare providers. This may be due to the presence of an outpatient-based smoking cessation treatment program at the VHA, which was a main difference between smoking cessation services offered between these healthcare systems. However, healthcare providers in both healthcare systems had the option of referring smokers to a quitline (the state quitline for AHC healthcare providers; either the state quitline or an internal VHA quitline for VHA healthcare providers). These results suggest that addressing facilitators and barriers to smoking cessation treatment at the healthcare system level may inform future implementation of smoking cessation treatment in diverse settings. For example, recent evidence demonstrates that system-level interventions such as implementation of electronic referral capability to the quitline (eReferral) can result in markedly increased rates of referral [28, 29].

The finding that 90% of healthcare providers overall performed evidence-based smoking cessation treatment, consisting of counseling plus smoking cessation medications or a referral to smoking cessation services, suggests far higher compliance than has been found in past research [6, 7]. A previous study found 16–64% of healthcare providers self-report “assisting” individuals to quit smoking [7] and another more recent study from 2015 found that 64% of healthcare providers reported assisting individuals in quitting smoking more than 75% of the time within the past 6 months [30]. The National Ambulatory Medical Care Survey from 2001 to 2004 reviewed medical records for a sample of outpatient visits and found that 81% of smokers did not have any documentation of assistance with smoking cessation (i.e., counseling, smoking cessation medications, referrals) and 2% received a prescription for nicotine replacement therapy or bupropion [31]. Although these healthcare provider self-reported and retrospective rates are lower than what was found in the present study, our research operationalized evidence-based practices as occurring at *any time* over the past year. Thus, the outcome in the present study may have been an easier target for healthcare providers to achieve. Ideally, every identified tobacco user should be offered evidence-based care at every clinical encounter [5]. Future implementation research should focus on strategies to streamline the integration of evidence-based smoking cessation treatment into the delivery of routine medical care via offering of the 5 A’s-based counseling, FDA-approved smoking cessation medication, and referrals to smoking cessation programs within and outside of the medical center. Results also suggest that while provision of smoking cessation medication has been high at both the VHA and AHC, efforts to increase healthcare providers’ referral to smoking cessation services are warranted at the AHC, and system-level efforts to increase counseling for smoking cessation delivered as part of routine medical care are warranted at both the AHC and VHA.

We found that smoking cessation attitudes towards effectiveness at reducing cancer-related mortality were not associated with provision of smoking cessation treatment. This finding is in contrast to prior literature showing a strong relationship between healthcare providers’ attitudes towards smoking cessation treatment and their practices [7, 32, 33], and should be interpreted with caution [7, 32, 33]. However, it is possible that healthcare providers in this study who followed the standard of care to provide smoking cessation treatment did so in spite of their personal beliefs about its effectiveness.

We found that healthcare providers who practiced in hospital-based settings (compared to community-based settings) and those who reported a higher (compared to a lower) proportion of smokers in their clinical practice were more likely to provide evidence-based smoking cessation treatment. Unexpectedly, we also found that White healthcare providers compared to their non-White healthcare providers, were less likely to provide evidence-based smoking cessation treatment. While racial disparities have been documented among smokers in the receipt of smoking cessation treatment [34], racial disparities on the healthcare provider end have not been widely studied. This study was not designed to assess why the race of the healthcare providers would be associated with provision of smoking cessation treatment. Future research should confirm this finding in other healthcare provider populations and investigate potential reasons (e.g., implicit bias) [35] for any observed treatment differences as a function of racial discordance in the provider-patient dyad.

Since the 1980s, the VHA has implemented system-wide interventions to promote population-based smoking cessation treatment in the Veteran population whereas other healthcare systems’ interventions are more recent. The VHA’s multi-pronged initiatives have consisted of: (1) behavioral health smoking cessation outpatient programs that offer smoking cessation counseling, smoking cessation medications, and connection to quitlines, (2) policy changes that allow all types of healthcare providers to prescribe smoking cessation medications (as opposed to restricting these prescriptions to healthcare providers in the smoking cessation program), (3) policies that eliminate co-payments for attending smoking cessation programs and provide free to low-cost smoking cessation medications, (4) annual smoking cessation clinical reminders/prompts for primary care providers, (5) development of VHA guidelines by clinical experts, (6) quality metrics that are routinely followed by VHA leadership, (7) healthcare provider incentives to perform smoking cessation treatment, (8) and integration of smoking cessation treatment into mental health treatment [13–16]. The VHA policy changes that expanded access to smoking cessation resources and encouraged healthcare providers to perform smoking cessation treatment led to an estimated 60% increase in prescription fills for smoking cessation medications from 2004 to 2008 [13]. These supportive system-level changes in the VHA likely explain the very high smoking cessation medication and referral rates among VHA healthcare providers in our study. The EHR at the AHC also supported healthcare providers’ provision of FDA-approved smoking cessation medication and referrals to the Tennessee state tobacco quitline. In contrast to the VHA, the AHC did not offer an outpatient (clinic-based) smoking cessation program. However, a robust inpatient service has existed since 2015. Certified smoking cessation counselors access a daily EHR-based list of current hospitalized smokers and approach them in an opt-out fashion, typically reaching about 10–20% of the high volume of smokers. The service also accommodates and prioritizes healthcare provider-based proactive consults (opt-in). This inpatient service was expanding during the time this study was conducted (February–May 2017), and was hospital-wide by the end of 2017. Despite electronic referrals to state quitlines and the recent implementation of a robust smoking cessation service for inpatients, only 61% of AHC healthcare providers (vs. 91% VHA) reported referring to these or other services over the past year. These comparatively low rates may reflect lower awareness of these resources among AHC healthcare providers and/or a lack of incorporation into healthcare providers’ daily workflow.

Future research and implementation efforts targeting smokers could explore these observed differences in smoking cessation treatment practices across healthcare systems in greater depth. Focus groups or interviews could assist in identifying strategies to integrate smoking cessation treatment into standard workflows. Effective strategies include healthcare provider-focused interventions, such as the implementation of clinical reminders or training in effective communication strategies for smoking cessation counseling, or team-based care models to supplement patient-physician interactions. For example, since this study was conducted, the AHC cancer center began offering comprehensive smoking cessation inpatient and outpatient services to smokers with cancer through a Cancer Moonshot program, the Cancer Center Cessation Initiative (C3I), launched by the National Cancer Institute (NCI) in 2017, which aims to assist NCI Designated Cancer Centers to enhance smoking cessation treatment of patients in cancer care [36]. Some services, including a bidirectional e-referral to the state tobacco quitline and automated telephone follow-up encounters, are intended to maximize the service’s reach [21]. Other smoking cessation-related activities, including one-on-one phone counseling with certified smoking cessation treatment specialists, free FDA-approved smoking cessation medication to eligible smokers, and precision care based on nicotine metabolism, are intended to augment personalized tobacco treatment [37, 38].

This study has several limitations. First, we relied on healthcare provider self-reporting of smoking cessation treatment practices, which may be subject to social desirability and recall bias. Selection bias may have also occurred. Next, our questionnaire item referring to counseling did not differentiate counseling as part of “Assist” versus “Advise” in the 5 A’s or counseling as treatment in a more intensive behavioral intervention and healthcare providers could have interpreted this differently. In our definition of evidence-based smoking cessation treatment, we defined referral as being to a service (program or quitline) that offers both counseling and smoking cessation medication; the VHA smoking cessation program and the overwhelming majority of quitlines offer smoking cessation medications and counseling [14, 16, 39, 40]. Next, healthcare providers at the VHA and AHC differed across several dimensions, but our study was underpowered to adjust for additional baseline characteristics other than medical specialty in our model testing the association between healthcare system and evidence-based smoking cessation treatment. It is possible that some of the differences in other baseline characteristics, such as practice location, may have impacted the precision of our estimates [39]. Finally, the results of this study reflect one geographic region and may not be generalizable. However, these healthcare systems were located within “Tobacco Nation,” a region in the U.S. Midwest, Mid-South and surrounding areas that is characterized by above-average rates of smoking and smoking-related disease. It is especially important that future research illuminates opportunities and challenges to providing effective smoking cessation treatment to this population of smokers.

## Conclusions

VHA healthcare providers were more likely to self-report providing evidence-based smoking cessation treatment compared to AHC providers, regardless of their attitudes towards effectiveness of smoking cessation. These findings likely reflect the VHA’s system-wide interventions that promote smoking cessation treatment for the Veteran population. Healthcare systems’ prioritization of and investment in smoking cessation services is critical to improving healthcare providers’ adherence to recommended smoking cessation treatment guidelines.

## Supplementary Information

**Additional file 1: Supplemental Table 1.** Questionnaire Items. **Supplemental Table 2.** Characteristics of Study Non-Responders.

## Data Availability

The dataset used in this study is available from the corresponding author upon reasonable request.

## Abbreviations

AHC: Academic health center
aOR: adjusted odds ratio
CI: Confidence interval
EHR: Electronic health record
FDA: Food and Drug Administration
IQR: Interquartile range
NCI: National Cancer Institute
U.S.: United States
VA: Veterans Affairs
VHA: Veterans Health Administration
5 A’s: Ask, Advise, Assess, Assist, Arrange
