# Supplementary material for: Evidence-based smoking cessation treatment: a comparison by healthcare system
Source: BMC Health Serv Res. 2021 Jan 7;21:33. doi: 10.1186/s12913-020-06016-5 (PMC7792006; doi:10.1186/s12913-020-06016-5)
Supplement: Supplementary file 1 — Additional file 1: Supplemental Table 1. Questionnaire Items. Supplemental Table 2. Characteristics of Study Non-Responders. [file 12913_2020_6016_MOESM1_ESM.docx]

Appendix

Supplemental Table 1: Questionnaire Items

| **Item Stem** | **Item Response Options** |
| --- | --- |
| Demographics |  |
| Do you provide any healthcare services to patients over age 50?  (eligibility criteria) | Yes  No (questionnaire end) |
| Have you provided healthcare services in the past 12 months?  (eligibility criteria) | Yes  No (questionnaire end) |
| What is your medical position?  (eligibility criteria) | Attending  Fellow/Resident/Intern  Physician Assistant  Nurse Practitioner  Other (questionnaire end) |
| What is your current year in training?  (if selected “Fellow/Resident/Intern”) | PGY 1  PGY 2  PGY 3  PGY 4  > PGY 4 |
| How many years have you been practicing as an attending, physician  assistant, or nurse practitioner?  (if selected “Attending”, “Physician Assistant”, or “Nurse Practitioner”) | Less than 1 year  1-5 years  6-10 years  11-20 years  > 20 years |
| What best describes your primary field of practice? | Internal Medicine  Internal Medicine-Hospitalist  Internal Medicine-Subspecialist  Obstetrics and Gynecology  Other |
| Within Internal Medicine, what best describes your primary field of practice?  (if selected “Internal Medicine”) | Family Medicine  General Internal Medicine/Primary Care  Geriatrics  Medicine-Pediatrics  Other |
| Which subspecialty best describes your primary field of practice?  (if selected “Internal Medicine-Subspecialty”) | Pulmonology  Hematology/Oncology  Other specialty |
| What percentage of your time is spent providing direct patient care (including  all time in your personal practice and supervising residents/fellows)? | < 25%  25-50%  51-75%  > 75% |
| Where is the location of your Vanderbilt practice? | Main campus  Community Clinic  Other |
| Where is the location of your VA practice? | Alvin C. York campus  Nashville campus  Community Based Outpatient Clinic or Annex  Other |
| Approximately, what percentage of your patients in your practice are current or  former smokers? | Current smokers: (free text)  Former smokers: (free text) |
| What is your gender? | Male  Female |
| Do you consider yourself to be… | Hispanic or Latino  NOT Hispanic or Latino |
| Which of the following best describes your race? Please mark all that apply. | White  Black or African American  Asian  Native Hawaiian/Other Pacific Islander  American Indian/Alaskan Native  Other |
| Smoking Cessation Practices |  |
| During the past 12 months, for an asymptomatic, high-risk patient, did you ever  provide smoking cessation services (counseling and/or medication)? | Yes  No  Don’t know |
| What type of smoking cessation services did you provide?  (if answered “yes” to previous item)   1. Referral for smoking cessation services 2. Smoking cessation counseling using the 5 A’s (Ask, Advise, Assess, Assist, Arrange) 3. FDA-approved smoking cessation medications (nicotine replacement, varenicline, buproprion) | Yes  No  Don’t know |
| Smoking Cessation Attitudes Towards Effectiveness |  |
| How effective or ineffective do you believe smoking cessation is in  reducing cancer-related mortality in the average, healthy individual for whom it  is recommended? | Very Effective  Moderately Effective  Minimally Effective  Not Effective  Don’t know |

Supplemental Table 2: Characteristics of Study Non-Responders

| **Characteristics** | **Non-Responders**  **N = 218 (%)** | **Total Analyzed Sample**  **N = 366 (%)** |
| --- | --- | --- |
| Gender, n (%) |  |  |
| Female | 119 (54.6) | 206 (56.3) |
| Medical Position, n (%) |  |  |
| Attending | 133 (61.0) | 171 (46.7) |
| Physicians in Training | 47 (21.6) | 159 (43.4) |
| Nurse Practitioner/Physician Assistant | 35 (16.1) | 36 (9.8) |
| Primary Field of Practice, n (%) |  |  |
| General Internal Medicine | 132 (60.6) | 215 (58.7) |
| Pulmonology | 40 (18.3) | 39 (10.7) |
| Hematology/Oncology | 25 (11.5) | 69 (18.9) |
| Gynecology | 18 (8.3) | 43 (11.7) |
| Practice Location, n (%) |  |  |
| Hospital-based clinic | 152 (69.7) | 262 (71.6) |
| Community-based clinic | 63 (28.9) | 104 (28.4) |
| Healthcare System, n (%) |  |  |
| Academic Health Center | 140 (65.6) | 273 (74.6) |
| Veterans Health Administration | 78 (35.8) | 93 (25.4) |

SupplementalTable 2 Legend: *VHA: Veterans Health Administration
